# Single-cell analysis of human airway epithelium identifies cell-type-specific responses to *Aspergillus* and *Coccidioides*

**DOI:** 10.1128/mbio.02121-25

**Published:** 2025-10-13

**Authors:** Alfred T. Harding, Arianne J. Crossen, Yanting Zhang, Jennifer L. Reedy, Kyle J. Basham, Olivia W. Hepworth, Viral S. Shah, Hannah Brown Harding, Manalee V. Surve, Patricia Simaku, Geneva N. Kwaku, Kirstine Nolling Jensen, Yohana Otto, Rebecca A. Ward, George R. Thompson, Bruce S. Klein, Jayaraj Rajagopal, Pritha Sen, Adam L. Haber, Jatin M. Vyas

**Affiliations:** 1 Institute for Medical Engineering and Sciences, Massachusetts Institute of Technology 2167, Cambridge, Massachusetts, USA; 2 Department of Microbiology, Harvard Medical School, Cambridge, Massachusetts, USA; 3 Division of Infectious Diseases, Department of Medicine, Massachusetts General Hospital 2348, Boston, Massachusetts, USA; 4 Department of Environmental Health, Harvard T. H. Chan School of Public Health 218854, Boston, Massachusetts, USA; 5 Department of Medicine, Harvard Medical School 1811 , Boston, Massachusetts, USA; 6 Harvard Stem Cell Institute 458677, Cambridge, Massachusetts, USA; 7 Center for Regenerative Medicine, Massachusetts General Hospital 2348, Boston, Massachusetts, USA; 8 Division of Pulmonary and Critical Care Medicine, Department of Medicine, Massachusetts General Hospital 2348, Boston, Massachusetts, USA; 9 Klarman Cell Observatory, Broad Institute of Massachusetts Institute of Technology and Harvard, Cambridge, Massachusetts, USA; 10 Broad Institute of MIT and Harvard, Cambridge, Massachusetts, USA; 11 Division of Infectious Diseases and Departments of Internal Medicine and Medical Microbiology and Immunology, University of California-Davis, Sacramento, California, USA; 12 Department of Pediatrics, School of Medicine and Public Health, University of Wisconsin-Madison 5228, Madison, Wisconsin, USA; 13 Department of Medicine, School of Medicine and Public Health, University of Wisconsin-Madison 5228, Madison, Wisconsin, USA; 14 Department of Medical Microbiology and Immunology, School of Medicine and Public Health, University of Wisconsin-Madison 5228, Madison, Wisconsin, USA; 15 Transplant, Oncology, and Immunocompromised Host Group, Division of Infectious Disease, Department of Medicine, Brigham and Women’s Hospital, Boston, Massachusetts, USA; 16 Dana-Farber Cancer Institute 1855, Boston, Massachusetts, USA; Hebrew University of Jerusalem Robert H Smith Faculty of Agriculture Food and Environment, Rehovot, Israel

**Keywords:** invasive fungal infection, *Aspergillus*, *Coccidioides*, airway epithelial cells, host response, lung infection

## Abstract

Respiratory fungal infections pose a significant threat to human health. Animal models do not fully recapitulate human disease, necessitating advanced models to study human-fungal pathogen interactions. In this study, we utilized primary human airway epithelial cells (hAECs) to recapitulate the lung environment *in vitro* and investigate cellular responses to two diverse, clinically significant fungal pathogens, *Aspergillus fumigatus* and *Coccidioides posadasii*. To understand the mechanisms of early pathogenesis for both fungi, we performed single-cell RNA sequencing of infected hAECs. Analysis revealed that both fungi induced cellular stress and cytokine production. However, the cellular subtypes affected and specific pathways induced differed between fungi, with *A. fumigatus* and *C. posadasii* triggering protein-folding-related stress in ciliated cells and hypoxia responses in secretory cells, respectively. This study represents one of the first reports of single-cell transcriptional analysis of hAECs infected with either *A. fumigatus* or *C. posadasii*, providing a vital data set to dissect the mechanism of disease and potentially identify targetable pathways.

**IMPORTANCE:**

Fungal infections in the lungs are lethal complications for those with compromised immune systems and have limited treatment strategies available. These options are restricted further by the increased prevalence of treatment-resistant fungi. Many studies focus on how our immune systems respond to these pathogens, yet airway epithelial cells remain an understudied component of fungal infections in the lungs. Here, the authors provide a transcriptional analysis of primary human airway epithelial cells stimulated by two distinct fungal pathogens, *Aspergillus fumigatus* and *Coccidioides posadasii*. These data will enable further mechanistic studies of the contribution of the airway epithelium to initial host responses and represent a powerful new resource for future investigations.

## INTRODUCTION

Pulmonary fungal infections are dreaded, particularly in immunocompromised individuals, due to high mortality rates and limitations in treatment strategies (1, 2). We investigated two clinically relevant fungal pathogens, *Aspergillus fumigatus* and *Coccidioides posadasii*, as they are both pulmonary pathogens but have different cell wall compositions, life cycles, and pathogenic strategies. Importantly, the two pathogens are clinically distinct. *A. fumigatus* is ubiquitous in the environment, making immunocompromised patients (e.g.*,* lung transplant or allogenic bone marrow transplant recipients) vulnerable to invasive aspergillosis. The mortality from *A. fumigatus*-related pulmonary disease remains unacceptably high (58%), and rates of *Aspergillus* multidrug resistance continue to increase (3–5). Inhaled conidia germinate into hyphae, a strong virulence trait. This change in morphotype dramatically changes the cell wall composition, exposing more antigenic carbohydrates like β-1,3 glucan and galactomannan when the rodlet and melanin layers are shed. Insights into the mechanisms that lung epithelial cells employ to direct immune cells against this dangerous fungus are thus clinically relevant and will guide new therapeutic strategies.

The dimorphic fungus *C. posadasii* causes coccidioidomycosis and is endemic to the southwestern US, Mexico, and South America, with the historical endemic boundaries expanding with climate change (6, 7), increasing the number of humans and animals at risk of exposure (8–10). Unlike *Aspergillus*, *C. posadasii* produces infection in both immunocompetent and immunocompromised individuals, implying that *C. posadasii* can thwart host defense mechanisms that *A. fumigatus* cannot and that distinct mechanisms are involved in controlling these infections (11, 12). Only 40% of *Coccidioides* infections are symptomatic, and these patients often present with acute or progressive self-limiting pneumonia, including the formation of pulmonary nodules (localized disease). *Coccidioides* is inhaled as arthroconidia, which convert into spherules that release endospores. *Coccidioides* differs from *Aspergillus* in virulence and dose required to cause infection. *Coccidioides* causes disease with inhalation of 1–50 arthroconidia in immunocompetent mice (13), whereas *Aspergillus* requires ~10^4^–10^5^ CFU to cause disease in immunocompromised (but not immunocompetent) patients and mice, again implying differences in host recognition and responses to these distinct fungal pathogens (14). We hypothesize that these differences provoke distinct and cell-type-specific epithelial transcriptional signatures from human lung epithelium that direct the ensuing immune response.

To elucidate the cellular responses and pathogen-specific mechanisms of these pulmonary fungal infections, we utilized human airway epithelial cells (hAECs) as an *in vitro* model to recapitulate the lung epithelium. The hAECs form a pseudostratified epithelial layer, closely mimicking the cellular environment of the human airway (15). In this study, hAECs were cultured and infected with either *A. fumigatus* or *C. posadasii*. Following infection, we isolated the cells and performed single-cell RNA sequencing (scRNA-seq) to obtain a high-resolution view of the transcriptional responses of specific cell types comprising the lung epithelium (16).

This approach enabled us to unveil both common and unique cell-specific responses and stress pathways activated by *A. fumigatus* and *C. posadasii* infection. Our findings revealed that *A. fumigatus* infection primarily affected ciliated cells, inducing protein-folding-related stress, whereas *C. posadasii* infection triggered a hypoxia response in secretory cells, coupled with increased cytokine expression. The distinct cellular responses to *A. fumigatus* and *C. posadasii* infections provide a valuable data set for understanding the mechanisms of pulmonary fungal diseases and identifying potential therapeutic targets.

## RESULTS

### Experimental design and hAEC differentiation and characterization

**Fig 1:**
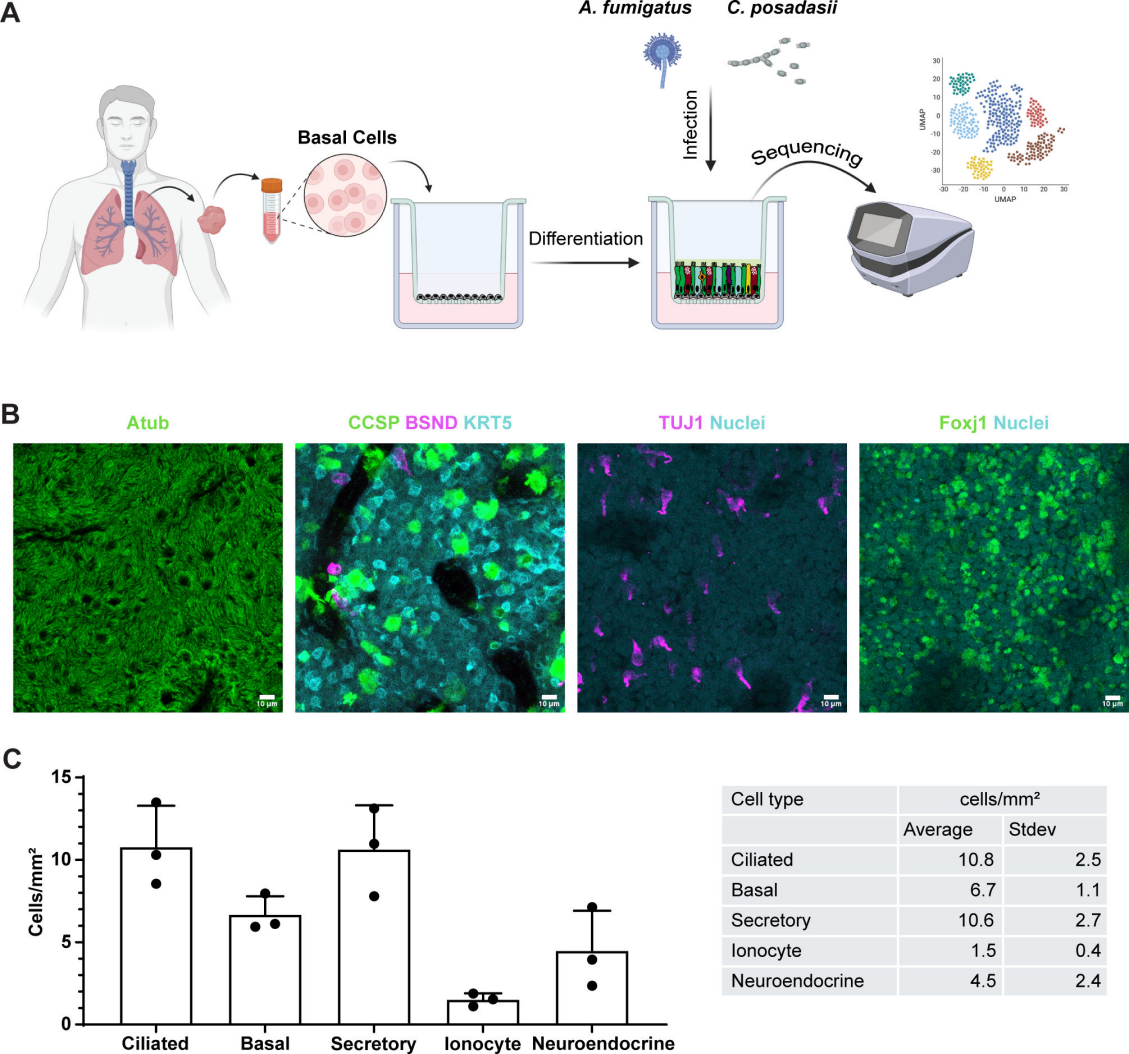
Experimental design and generation of hAEC models. (**A**) A schematic outlining the experimental design wherein airway basal cells are isolated from human volunteers, expanded, and differentiated, and then infected with either *A. fumigatus* or *C. posadasii* before undergoing scRNA-seq. (**B**) Representative images of fully differentiated hAECs. Cell markers for ciliated cells (AcTub or Foxj1), basal stem cells (KRT5), club cells (CCSP), ionocytes (BSND), and neuroendocrine (TUJ1) were used. Size bar = 20 µm. (**C**) Quantification of epithelial subtypes per mm^2^ from images captured in panel **B**.

To better understand the mechanisms by which fungal pathogens infect and cause disease in the lung, we designed a study wherein we generated *ex vivo* human lung epithelia from basal cells isolated from the same donor. Following differentiation of hAECs, we infected them with either *A. fumigatus* or *C. posadasii* and then subsequently performed scRNA-seq (Fig. 1A). To create a robust *in vitro* model of the human airway epithelium, we began by isolating basal cells from human donor lung tissues. These primary basal cells were cultured according to previously published protocols (17, 18). Briefly, we expanded cells in small-airway epithelial cell medium (SAGM) supplemented with various growth factors and inhibitors to promote their expansion (details are provided in the Materials and Methods section). Basal cells were seeded onto Transwell inserts to establish an air-liquid interface (ALI) culture, which was maintained for approximately 23 days. During this period, the basal cells differentiate into a pseudostratified epithelium that mimics the cellular composition and architecture of the human airway (19, 20).

To confirm that our differentiation was successful, we fixed and stained hAECs in a subset of Transwells grown in parallel to ensure the major airway cell subtypes were present. Specific antibodies were used to stain for basal cells (KRT5), club cells (SCGB1A1/club cell secretory protein [CCSP]), ciliated cells (acetylated tubulin/Foxj1), ionocytes (BSND), and neuroendocrine (TUJ1) (20–23). The immunofluorescent images illustrate the successful establishment of a pseudostratified airway epithelium, comprising key cell subtypes of the human airway, signaling that our model had representative cell types at predicted proportions (24–27) (Fig. 1B and C).

### Infection of differentiated airway epithelium with *A. fumigatus* and *C. posadasii*

Once validated, our hAECs were infected with *A. fumigatus* or *C. posadasii* to investigate the response of the differentiated airway epithelium to infection. Each ALI culture was incubated with 10^7^/cm^2^ fungal arthroconidia of *A. fumigatus* or *C. posadasii* for 6 or 18 h, respectively. A shorter infection period was required for *A. fumigatus* due to its hyphal formation and subsequent damage to AECs, which makes the isolation of single cells difficult (28) and the kinetics of cytokine responses seen in hAECs (18). We selected 18 hours for *C. posadasii* to capture robust transcriptional responses during early infection stages. While conversion of arthroconidia to hyphal forms may occur at this timepoint, we intended to focus on the host response to the initial phases of fungal exposure, which are most relevant to inhalation and early mucosal interaction *in vivo*. Single-cell suspensions from the *in vitro*-infected hAEC cultures were used to load individual channels on the 10× Chromium Controller, and gene expression libraries were subsequently created using the 3′ V3.1 chemistry (29). Pooled libraries were sequenced at a depth of 9,751 or 11,059 reads per cell for *A. fumigatus* vs. mock, respectively. For the *C. posadasii* experiment, the depth sequences were 6,180 or 6,531 reads per cell for infected vs. mock, respectively.

**Fig 2:**
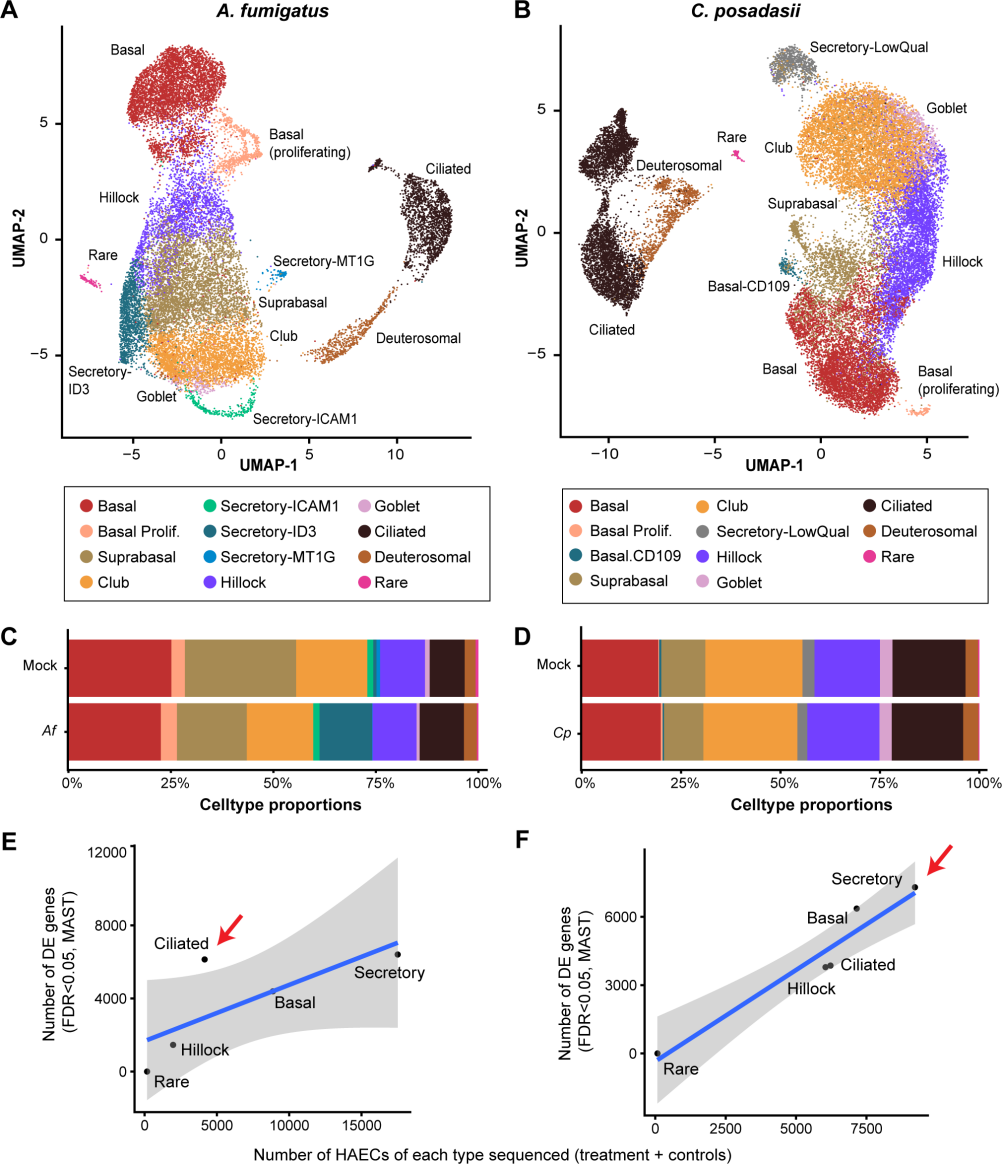
Cell clustering and differential gene expression of scRNA-seq following hAEC infection. UMAP embeddings of 20,560 (hAECs, points) infected with *A. fumigatus* (10^7^ conidia, B5233 strain) (**A**) or 28,724 (hAECs, points) infected with *C. posadasii* (10^7^ arthroconidia, Silveira strain) (**B**) and uninfected controls (mock). Points are colored by assignment to cell types using unsupervised clustering with the Leiden algorithm. Cell type proportions identified during UMAP generation during *A. fumigatus* (*Af*) (**C**) or *C. posadasii* (*Cp*) (**D**) infection as compared to mock. Scatter plots show the relationship between the number of hAECs of each cell type in the scRNA-seq analysis (*x*-axis) and the number of differentially expressed genes (DEGs; false discovery rate [FDR] < 0.05) between *A. fumigatus* (**E**) or *C. posadasii* (**F**) and uninfected controls. Blue line: linear model fit, shaded area 90% confidence interval. Red arrows indicate the cell subtype with the highest DEGs. *n* = 1.

The sequencing data from lung epithelium of both *A. fumigatus* and *C. posadasii*-infected samples were analyzed using unsupervised clustering, which allowed us to identify and categorize the relevant cell types and visualize them using uniform manifold approximation and projection (UMAP) (Fig. 2A and B). These UMAP plots highlighted the distinct cell populations of the airway epithelium, including basal, club, ciliated, hillock, goblet, and other rarer epithelial subtypes (e.g.*,* ionocytes, tuft, and neuroendocrine cells). The proportion distribution of each cell type was quantified and found to be similar to reported numbers from human airways (24–27), providing further support for the validity of our model (Fig. 2C and D).

We then examined the number of differentially expressed genes (DEGs) during infection compared to mock-treated cells. In both infections, we observed little in the way of DEGs in our rare cell subtypes (i.e.*,* ionocytes, neuroendocrine, and tuft cells) because of the low number of hAECs detected in those groups, limiting our statistical power (Fig. S1). All other cell types, however, displayed a multitude of DEGs. Our analysis revealed that *A. fumigatus* infection appeared to most dramatically impact ciliated cells, significantly (false discovery rate [FDR] <0.05) altering the expression of greater than 5,000 genes (Fig. 2E), whereas *C. posadasii* infection displayed the greatest effect on secretory cells (goblet and club cells), inducing significant changes in over 6,000 genes (Fig. 2F). This differential response underscores potentially unique cellular targets and mechanisms employed by each pathogen during infection.

### Identification of upregulated genes in the respiratory epithelium with *A. fumigatus* stimulation

**Fig 3:**
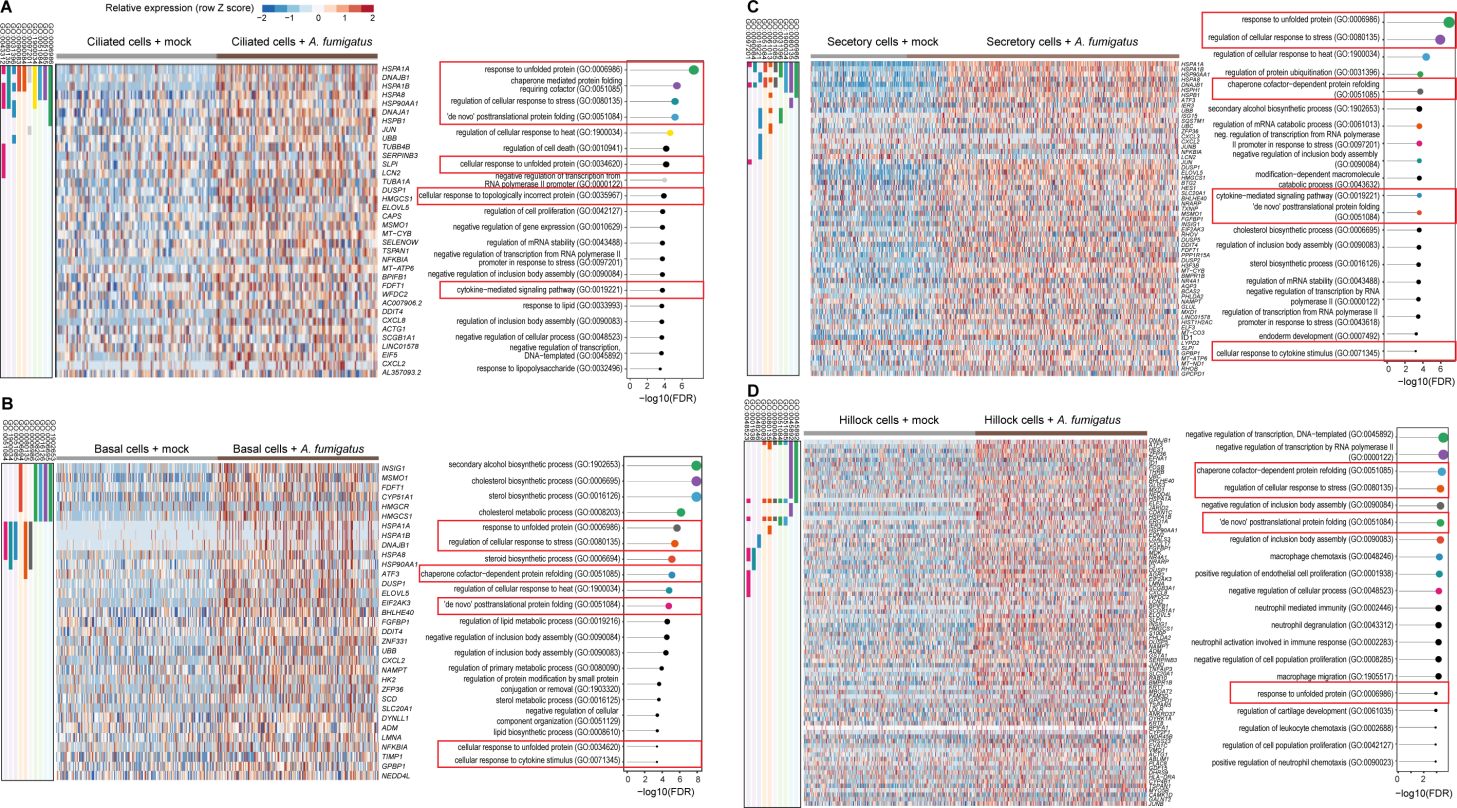
Cell-specific transcriptional programs activated in hAECs by *A. fumigatus*. Top upregulated (FDR < 0.05) genes and gene ontology (GO) biological process pathways in human airway cells (right columns) by infection with *A. fumigatus*. Ciliated cells (**A**), basal cells (**B**), secretory cells (**C**), and hillock cells (**D**) are detailed. Individual gene membership in pathways is indicated in the color legend, left. Relevant GO pathways are highlighted by the red boxes.

To understand the transcriptional response to *A. fumigatus* by cell type, we analyzed the single-cell transcriptome of basal, hillock, secretory, and ciliated cells as distinct clusters. These upregulated genes were visualized using heatmaps, and gene set enrichment analyses were conducted to explore their functional associations (Fig. 3). In basal cells (Fig. 3B), we observed that genes involved in metabolic biosynthetic pathways were potently induced. In contrast, hillock cells (Fig. 3D) induced genes involved in the negative regulation of key proteins involved in transcription. Both the transcriptional response in secretory (Fig. 3C) and ciliated cells (Fig. 3A) were dominated by genes involved in the unfolded protein response (UPR). High levels of upregulated genes related to sterol biosynthesis were identified in basal and secretory cells, which may be associated with the UPR or induction of cell death (Fig. 3B and C).

### Analysis of ciliated cells during *A. fumigatus* infection

Multiple diseases that carry an elevated risk of *A. fumigatus* infection demonstrate altered status in ciliated cells. For example, individuals with chronic obstructive pulmonary disease, asthma, and cystic fibrosis display epithelial goblet cell hyperplasia, which skews the ratio of epithelium toward more secretory cells at the expense of ciliated cells (30–32). Additionally, respiratory viral infections associated with *A. fumigatus* superinfections (e.g.*,* SARS-CoV-2 and influenza) are linked to the shedding of ciliated airway epithelial cells, reduced mucociliary clearance, and static cilia (33). Given that ciliated cells were the most impacted cell type during *A. fumigatus* infection, in addition to the biological relevance in high-risk patients, we performed a detailed analysis of the gene expression profile of these cells compared to mock-treated controls (Fig. 3A). Top DEGs were identified using pairwise comparisons with the MAST test. We applied thresholds of a minimum UMI count of 0 and expression in more than 10 cells. The genes were further filtered based on a log_2_ fold change greater than 0.25 and an adjusted *P*-value (FDR) of less than 0.05. These genes were then visualized using heatmaps, and enrichment analysis was conducted to explore their functional associations. We examined the top-upregulated genes in *A. fumigatus-*infected ciliated cells and their corresponding pathways (Fig. 3A).

Our analysis revealed a strong enrichment for stress response genes, particularly those associated with the UPR. Notably, the genes encoding heat shock proteins such as HSPA1A, HSPA1B, HSPA8, HSP90AA1, DNAJB1, HSPB1, and DNAJA1 were all strongly upregulated. Many of these cytoplasmic chaperones are transcriptionally regulated by the heat shock factor 1 (HSF1) protein, which is sequestered by HSP90 until unfolded proteins compete for HSP90, allowing HSF1 release and non-canonical UPR activation (34). Additionally, the gene *DDIT4*, which is involved in cellular stress response and cell survival, was also highly expressed following stimulation with *A. fumigatus*. This indicates that *A. fumigatus* infection triggers a robust UPR in ciliated cells, likely as a defense mechanism against the pathogen-induced cellular stress. Moreover, genes encoding key cytokines (i.e.*,* CXCL8, CXCL2) and *NFKBIA* were elevated in infected ciliated cells.

### Identification of upregulated genes in the respiratory epithelium with *C. posadasii* stimulation

**Fig 4:**
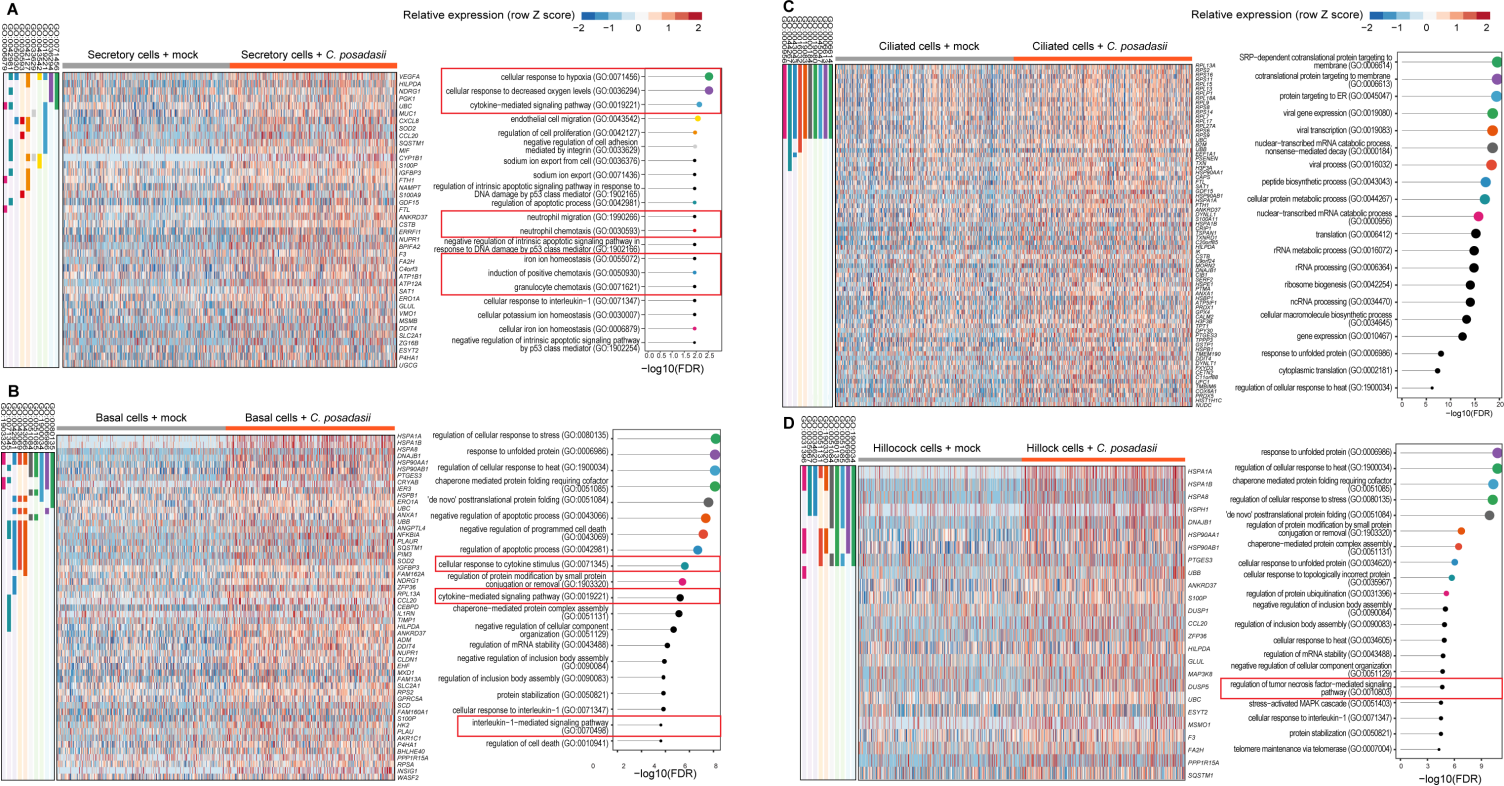
Cell-specific transcriptional programs activated in hAECs by *C. posadasii*. Top upregulated (FDR < 0.05) genes and gene ontology (GO) biological process pathways in human airway cells (right columns) by infection with *C. posadasii*. Secretory cells (**A**), basal cells (**B**), ciliated cells (**C**), and hillock cells (**D**) are detailed. Individual gene membership in pathways is indicated in the color legend, left. Relevant GO pathways are highlighted by the red boxes.

Similar to *A. fumigatus*, we analyzed the single-cell transcriptome of basal, hillock, secretory, and ciliated cells of *C. posadasii*. These upregulated genes were visualized using heatmaps, and enrichment analyses were conducted to explore their functional associations (Fig. 4). Interestingly, the UPR dominated two cell types—hillock (Fig. 4D) and basal cells (Fig. 4B), which is in sharp contrast to *A. fumigatus*-stimulated cells, in which secretory (Fig. 4A) and ciliated cells (Fig. 4C) had the UPR as the dominant pathway affected. For *C. posadasii*-stimulated ciliated cells, we observed a potent upregulation of genes involved in protein targeting to membranes, while secretory cells, when stimulated by *C. posadasii*, demonstrated increased transcription of genes involved in the hypoxia response and immune cell chemotaxis (Fig. 4).

### Analysis of secretory cells during *C. posadasii* infection

Club cells line most of the mucosa, making up 60% of the epithelial lining (35). One study revealed that *Coccidioides* perturbed surfactant proteins A and D during infection, which are mainly synthesized by type II alveolar cells and club cells (36). Prior studies have implicated these secretory cells as important in the regulation of innate resistance to another dimorphic fungi, *Blastomyces*, by releasing products such as CCL2, CCL20, and IL-1 to assemble myeloid and lymphoid cells to contain the fungus (37). Given the large amount of DEGs in epithelium exposed to *C. posadasii*, we focused on the secretory cells for further analysis. We next performed a detailed examination of the transcriptional changes induced by *C. posadasii* infection in secretory cells (Fig. 4) and identified enriched pathways among the top genes upregulated during *C. posadasii* infection (Fig. 4A). Our findings revealed significant transcriptional changes activating the hypoxia response system and immune cell chemotaxis, both of which are hallmarks of *C. posadasii* infection in the lung (38).

**Fig 5:**
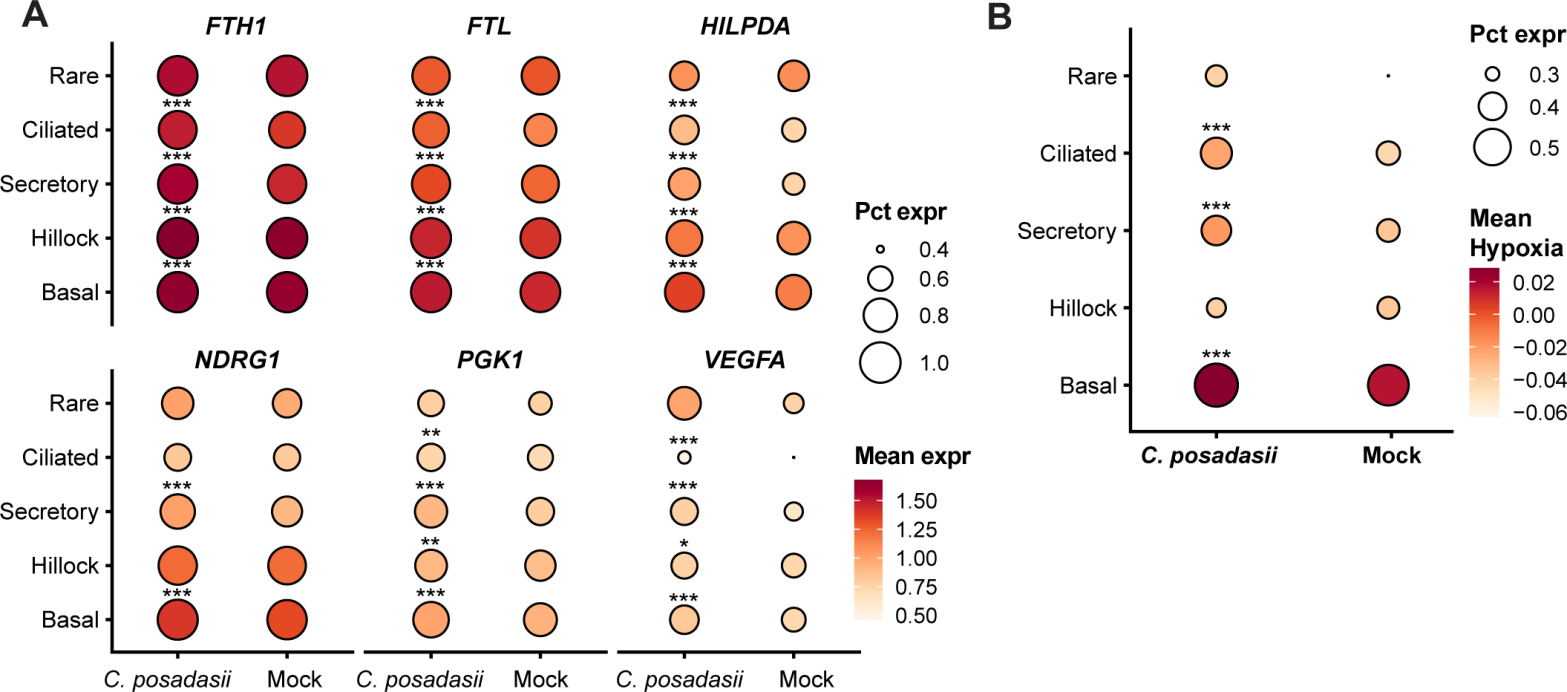
Transcriptional changes in hypoxia-related genes from *C. posadasii* stimulation of hAECs. (**A**). Dot plot for six hypoxia genes *FTH1, FTL, HILPDA, NDRG1, PGK1,* and *VEGFA* comparing uninfected vs. C. posadasii stimulated cells and segregated into rare, ciliated, secretory, hillock, and basal cells. (**B**). Distribution plots of hypoxia pathway scores across cell types. *, *P* < 0.05; **, *P* < 0.01; ***, *P* < 0.001. For both panels, the size and shading of the circles represent percent expression and mean expression, respectively.

Many top genes induced by *C. posadasii* are strongly involved in hypoxia and/or cellular stress, highlighting its importance during the early period of infection (Fig. 5A). Vascular endothelial growth factor A (VEGFA) is a critical regulator of angiogenesis and is typically upregulated under hypoxic conditions to promote blood vessel formation and increase oxygen supply (39), a pathway that is very important in the host response to other fungal pathogens (40, 41). Similarly, hypoxia-inducible lipid droplet-associated protein (HILPDA), N-myc downstream-regulated gene 1 (NDRG1), and phosphoglycerate kinase 1 (PGK1) are also associated with cellular adaptations to low oxygen levels, playing roles in lipid metabolism, stress response, and regulation of anaerobic metabolism, respectively (42–44).

Additionally, the genes FTL (ferritin light chain) and FTH1 (ferritin heavy chain 1) were significantly upregulated, indicating an involvement in ferroptosis and hypoxia. Ferritin is a key regulator of iron homeostasis and plays a role in protecting cells from oxidative stress by sequestering free iron, which can catalyze the formation of reactive oxygen species. The upregulation of FTL and FTH1 suggests a potential mechanism by which *C. posadasii* could be inducing hypoxic stress, namely the disruption of iron homeostasis. To be sure, the hypoxia gene induction signature was observed not only in secretory cells but was also found in basal and ciliated cells (Fig. 5B).

In addition to hypoxia and stress-associated genes, our analyses highlighted the upregulation of several immune-related genes, specifically those involved in regulating immune cell recruitment. Chemokines, including CXCL8, CCL20, and MIF, recruit immune cells, such as neutrophils, macrophages, and T cells, to sites of infection and inflammation and play critical roles in the immune response to pathogens (45–48). Furthermore, we identified two members of the S100 family, S100P and S100A9, among the top-induced genes in response to *C. posadasii*. The S100 family is a group of calcium-binding proteins that are strongly associated with inflammatory processes and immune cell chemotaxis. Interestingly, CCL20 and other CC chemokines, as well as S100 family members, are heavily induced by hypoxia pathways (49), suggesting a potential link between the specific hypoxic and immune responses induced by *C. posadasii*.

### Downregulated genes in airway cells stimulated by *A. fumigatus* or *C. posadasii*

**Fig 6:**
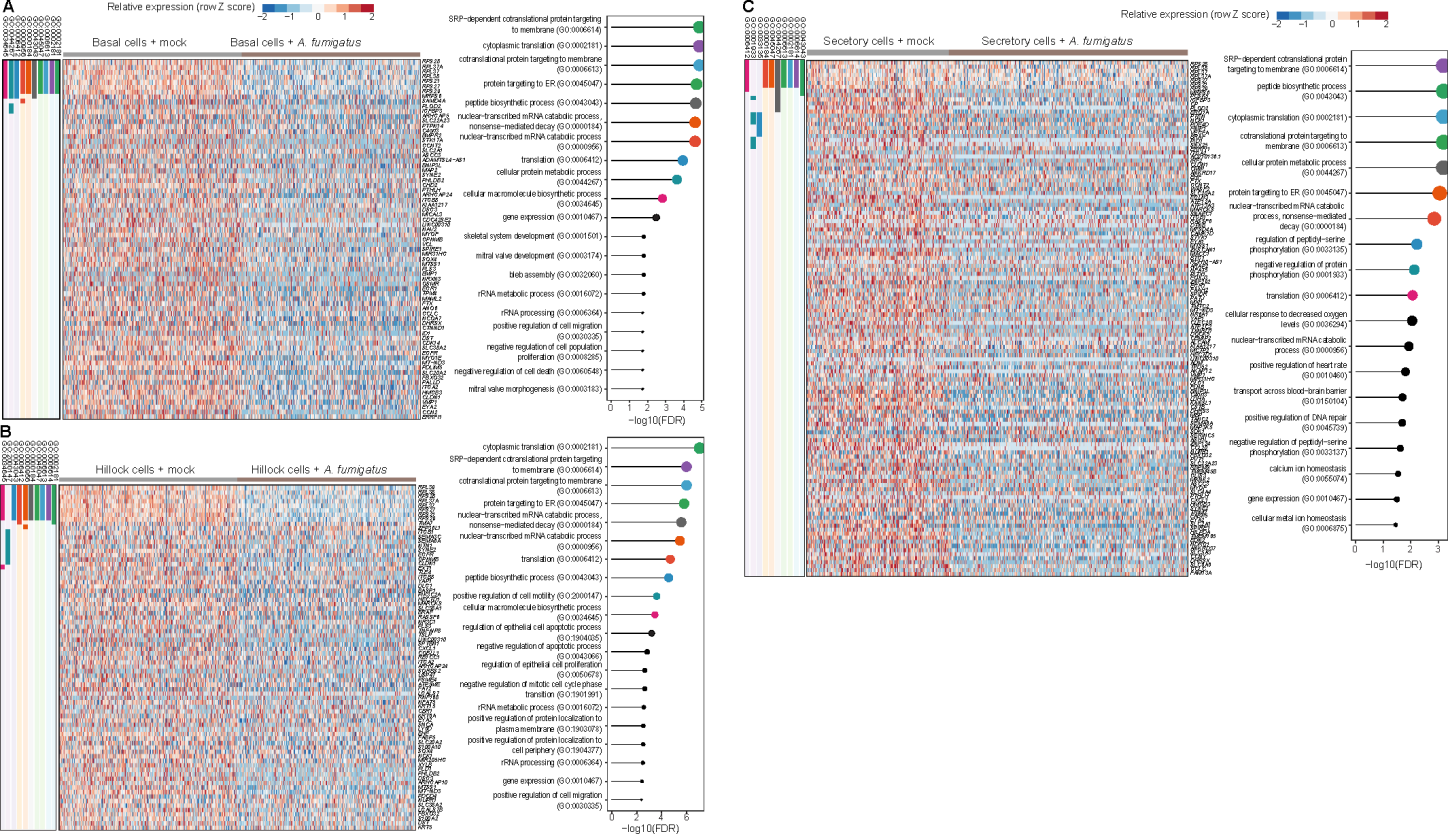
Cell-specific transcriptional programs downregulated in hAECs by *A. fumigatus*. Top upregulated (FDR < 0.05) genes and gene ontology (GO) biological process pathways in human airway cells (right columns) by infection with *A. fumigatus*. Basal cells (**A**), hillock cells (**B**), and secretory cells (**C**) are detailed. Individual gene membership in pathways is indicated in the color legend, left. Relevant GO pathways are highlighted by the red boxes.

**Fig 7:**
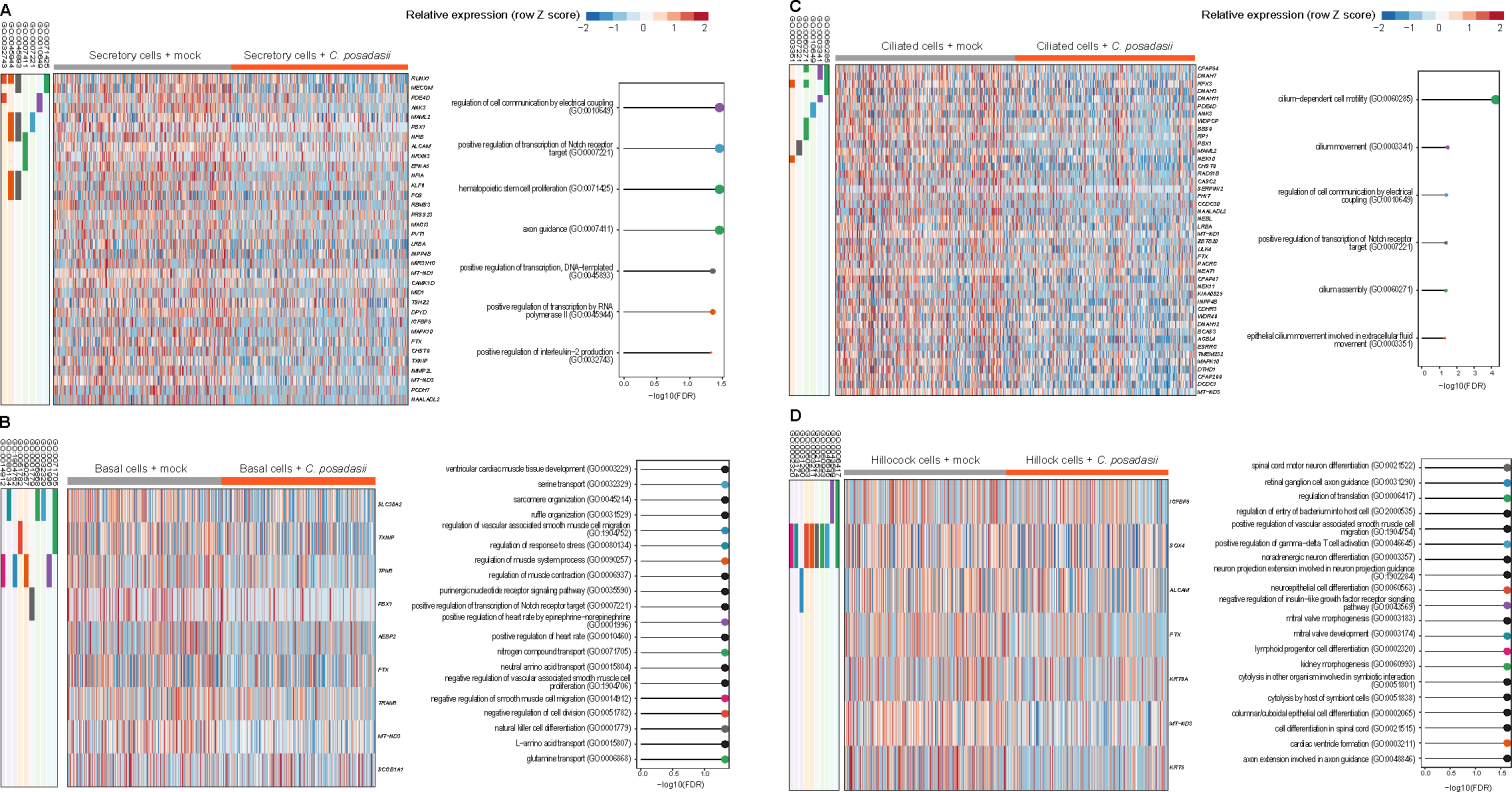
Cell-specific transcriptional programs downregulated in hAECs by *C. posadasii*. Top upregulated (FDR < 0.05) genes and gene ontology (GO) biological process pathways in human airway cells (right columns) by infection with *C. posadasii*. Secretory cells (**A**), basal cells (**B**), ciliated cells (**C**), and hillock cells (**D**) are detailed. Individual gene membership in pathways is indicated in the color legend, left. Relevant GO pathways are highlighted by the red boxes.

In addition to the examination of upregulated gene expressions in stimulated lung epithelial cells, our approach permits the interrogation of downregulated genes. In *A. fumigatus*, we observed that hillock cells had the greatest number of downregulated genes (Fig. 6). In sharp contrast, ciliated cells, which had the greatest number of upregulated genes, had no significant downregulated genes (Table S1). Within the hillock, basal and secretory cells, genes involved in protein translation and insertion of proteins into membranes demonstrated the most potent downregulation (Fig. 6A through C). For airway cells challenged by *C. posadasii*, both hillock and basal cells showed the greatest number of downregulated genes (Fig. 7). In contrast to *A. fumigatus*-stimulated cells, no dominant common gene pathway was identified in *C. posadasii*-stimulated cells. Secretory and ciliated cells showed only a handful of gene pathways downregulated, with no dominant pattern observed (Fig. 7A and C).

### Common and differential gene expression during *A. fumigatus* and *C. posadasii* infections

**Fig 8:**
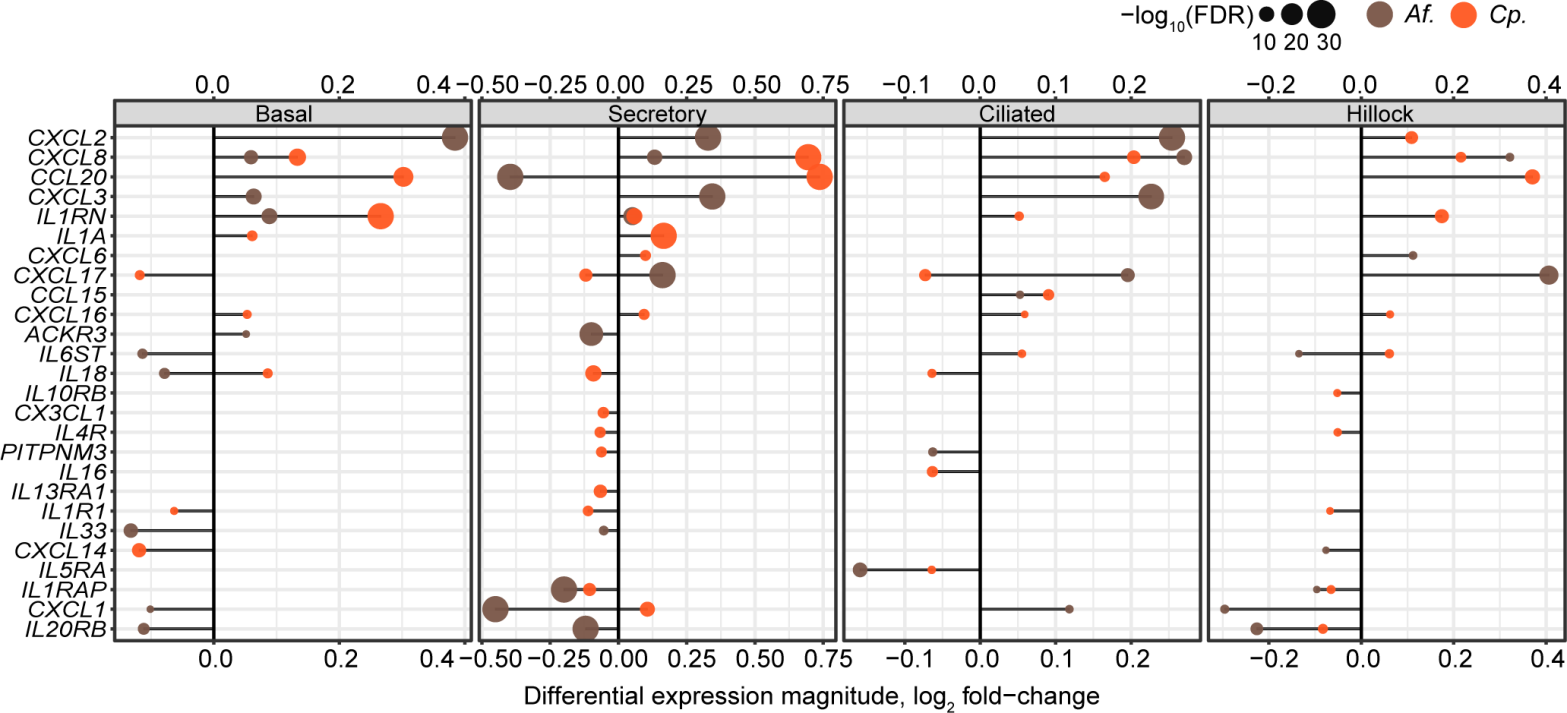
Cell-type-specific paracrine signaling responses to fungal pathogens. “Lollipop” plots show the relationship between differential expression effect size (*x*-axis) and significance (dot size, legend) for any chemokine, interleukin, or cognate receptor (rows, HUGO gene sets 189, 483, 601, and 602), by each fungal pathogen (color legend; *Af = A. fumigatus*; *Cp = C. posadasii*) for each indicated cell type.

Given the prevalence of immune modulators in both our *A. fumigatus* and *C. posadasii* gene lists, as well as the importance of immune cell recruitment for controlling and clearing fungal respiratory pathogens, we directly compared DEGs encoding paracrine signaling molecules induced by these two pathogens. Figure 8 illustrates the common and unique differential expression of cytokines and immune regulators across the major cell types (i.e*.,* basal, secretory, ciliated, and hillock) of our hAECs during infection with *A. fumigatus* and *C. posadasii*. CXCL8, the most potent human neutrophil-attracting chemokine, was the only chemokine robustly induced by both fungi, albeit to differing degrees depending on the cell subtype.

Both pathogens displayed robust signatures of cytokine expression involved in the regulation of immune cell recruitment, suggesting that the specific subsets of immune cells recruited might differ between the two fungi. *A. fumigatus* specifically upregulated *CXCL2* and *CXCL3,* in addition to *CXCL8*, in almost all cell types examined. Like CXCL8, both chemokines play significant roles in the recruitment and activation of neutrophils, which are essential for the early immune response to fungal infections. Given this unique signature for several key neutrophil-specific chemokines, our data suggest that the lung epithelium is a critical early initiator of the neutrophil response against *A. fumigatus*.

Unlike *A. fumigatus*, *C. posadasii* induced the expression of chemokines that were much less skewed toward neutrophil recruitment. CCL20, also known as MIP-3α, is a far less potent recruiter of neutrophils, instead displaying a stronger preference for lymphocytes and dendritic cells, helping develop adaptive immune responses at the site of infection as opposed to just innate immunity (46, 47, 50, 51). Curiously, we revealed the unique induction of both *IL1α* and *IL1RN* (interleukin 1 receptor antagonist) during *C. posadasii* infection. IL-1α is a potent pro-inflammatory cytokine that not only activates TNFα signaling but also recruits neutrophils to the site of fungal infection (49). IL1RN, however, functions as a direct antagonist to IL-1α, and based on our analysis, the gene is induced at higher levels than *IL1α*. These observations once again point to another piece of evidence where neutrophil recruitment to the lung is uniquely blunted during *C. posadasii* infection as compared to *A. fumigatus*. Overall, these data suggest that infection by *A. fumigatus* or *C. posadasii* does indeed induce unique inflammatory signatures in specific cell subtypes of the lung epithelia that likely impact the outcome and disease presentation.

## DISCUSSION 

In this study, we investigated the transcriptional responses of primary hAECs to infections by *A. fumigatus* and *C. posadasii*. By leveraging unbiased, single-cell sequencing approaches, we delineated the cellular and molecular alterations induced by these respiratory fungal pathogens, providing novel insights into the specific pathways and genes involved in the host response. Our results revealed both distinct and shared transcriptional changes elicited by *A. fumigatus* and *C. posadasii* infections. The induction of stress response genes, particularly those associated with the UPR, was a prominent feature of *A. fumigatus* infection. Notably, ciliated cells exhibited the highest number of DEGs, underscoring their vulnerability to fungal assault. *C. posadasii* exhibited a similar, but clearly distinct profile. Secretory cells, not ciliated cells, were most heavily impacted by *C. posadasii*, and the activated cellular stress pathways were strongly associated with hypoxia response as opposed to protein folding. Interestingly, the commonality of downregulation of protein translation and protein insertion into membrane pathways seen in *A. fumigatus-*stimulated lung epithelial cells was distinct when compared to *C. posadasii*-stimulated cells. No common theme was unveiled in the list of downregulated gene pathways for *C. posadasii*-stimulated lung epithelial cells. Overall, the initial analysis of the data set has already identified several interesting avenues of future research that could uncover key insights into the differing mechanisms of controlling and potentially treating these infections.

Perhaps the most obvious difference noted between epithelial immune responses generated to *Aspergillus* compared to *Coccidioides* infection was in chemokine expression. CXCL8 and CXCL2 are critical chemokines involved in neutrophil recruitment and activation, which play a vital role in the host’s pro-inflammatory immune response to fungal infections. While both pathogens displayed an induction of CXCL8, the strongest chemoattractant for neutrophils, *A. fumigatus* appeared to induce a stronger neutrophil recruitment signature than *C. posadasii,* as it uniquely induced CXCL2 and CXCL3. It should be noted that this increase in CXCL8 transcript may not correlate with the secretion of the chemokine, as we recently reported (18). It is well established that both *A. fumigatus* and *C. posadasii* induce neutrophil recruitment to the lung (17, 52–55), but to our knowledge, there has not been a direct comparison of the neutrophil-recruiting capabilities of the two. It is possible that *A. fumigatus* may be far more potent than *C. posadasii*, and perhaps this discrepancy in neutrophil recruitment explains why healthy patients are able to control *A. fumigatus* infection but not *C. posadasii*.

Innate responses must be tightly regulated to ensure that clearance of the invading pathogen does not lead to unnecessary host cell damage. *A. fumigatus* increased expression of *NFKBIA* encodes IκBα, an inhibitor of the NFκB pathway, in ciliated cells. This protein regulates inflammation and immune responses as well as cell survival, highlighting potential regulation of this complex immune response (56). Taken together with the increased expression of these cytokines, this suggests an active inflammatory response to *A. fumigatus* infection, aimed at controlling and clearing the pathogen.

Our data unveiled UPR pathways as highly changed in response to *A. fumigatus*, especially in ciliated cells, which supports prior murine studies showing increased UPR pathway components (57). UPR pathways are stress response pathways, particularly endoplasmic reticulum (ER) stress, and are involved in maintaining cellular homeostasis. ER stress has been implicated in lung diseases that predispose to *A. fumigatus* infection (58). The master regulators of the UPR are three transmembrane receptors in the ER, all of which were upregulated: (i) inositol-requiring enzyme 1 (IRE1α), (ii) activating transcription factor 6 (ATF6), and (iii) double-stranded RNA-activated protein kinase-like ER kinase (PERK) (59). The UPR and other stress responses link the folding of proteins to inflammatory signaling and cell survival, which is well adapted to the recognition of non-host cell proteins encoded by viruses and other pathogens. The synthesis of misfolded proteins encoded by microbial pathogens creates cell stress and inflammation. The UPR activates apoptosis and necrosis to remove infected epithelial cells and pathogens (59). Interestingly, UPR activation has been linked to calcium homeostasis. Although *Aspergillus*-mediated calcium signaling has been less studied in airway epithelium, numerous studies have shown an association in other host immune cells (60–64). Furthermore, we observed that *A. fumigatus* blunts calcium flux through melanin-mediated mechanisms in airway epithelium (18). It is possible that the UPR response triggered by *A. fumigatus* occurs in a calcium-dependent fashion.

Another interesting finding was the induction of hypoxia-associated genes by *C. posadasii,* which, to our knowledge, had not been directly shown in humans before, although one study implicated upregulation of HIF1α in *C. immitis*-resistant mice compared to susceptible mice (38) and a recent study in mice demonstrated changes to hypoxia-associated genes in response to *C. posadasii* (65). While we did not detect hypoxic signatures during *A. fumigatus* infection, others have reported it during lung infections at later timepoints than 6 h (66, 67). The current role in the creation of a hypoxic environment in the lung by fungal pathogens is unclear. Hypoxia regulates both angiogenesis and immune cell signaling/recruitment, vital pathways for controlling the dissemination and spread of the pathogen, as well as potentially regulating the fungal lifecycle (41). Fungal morphogenesis is known to be sensitive to oxygen tension, and hypoxic conditions have been implicated in modulating dimorphic switching and virulence gene expression in related fungal pathogens. Whether the observed hypoxia response reflects an active manipulation by the fungus or a consequence of its growth and metabolism remains to be determined. Furthermore, hypoxic respiratory failure is a rare but fatal condition that can develop during severe *Coccidioides* infections (68). It is also possible that the large-scale spreading of the pathogen throughout the lung and the induction of this signature contribute to the formation of this severe disorder.

Although this study provides a rich and novel data set that can be used to drive future investigations, there are limitations that should be noted. First, the differentiated epithelial cells were derived from one biological source. This sample does not allow for full representation of the population and does not enable the full appreciation of biological variables (i.e.*,* sex, race, ancestry, age) that may influence responses to fungal pathogens. For example, it is known that both pregnancy and exposure to air pollution can increase the risk of developing severe coccidioidomycosis (69–71). Furthermore, the sample provided came from a healthy individual with no evidence of lung disease or immunocompromised status. Given the high incidence and mortality rates of *Aspergillus* infections that occur in persons with pre-existing lung disease or immunologically fragile states (3–5), future research should explore how risk factors may influence cell-specific epithelial-mediated responses. Additionally, our small sample size limits our ability to examine changes in rarer cell populations. Another limitation is the differences in infection times and multiplicity of infection (MOI) for the two pathogens. We chose the two time points because prolonged time points beyond 6 h for *A. fumigatus* infection led to increased hyphal growth and epithelial death, whereas *C. posadasii* had much slower growth and allowed for a longer infection period. It is possible that some of our results contribute to the observed variations in cellular responses. If we were to use identical conditions for both microorganisms (e.g*.,* effector to target ratio, duration of exposure), we would not likely optimize for either host-pathogen combination. Lastly, our data only examine one strain of *Aspergillus* and *Coccidioides*. Different *A. fumigatus* strains display varied virulence rates and conidial surface proteins that may result in changes to the transcriptional responses in epithelial cells (72, 73). Furthermore, *Coccidioides immitis* is another species that results in human disease and requires further understanding (74). Regardless, future studies will focus on expanding the number of samples to truly appreciate the complexity of airway epithelial-mediated response to pulmonary fungal pathogens, as well as additional pulmonary fungal pathogens with varying times and MOI.

This data set and our analysis not only enhance our understanding of pulmonary fungal infections but also open new avenues for research into targeted therapies and interventions and provide opportunities for other investigators to explore pathways within human lung epithelial cells. These data, coupled with recent scRNA-seq data sets from mouse innate immune cells responding to *in vivo* challenge with *C. posadasii* (75, 76), will enable us to understand the pathogenesis of these invasive fungal diseases. Furthermore, these data provide an exciting new tool for the fungal field to expand its investigation. Future studies will delve deeper into the unexplored aspects of this data, potentially uncovering novel biomarkers and therapeutic targets to improve the management and treatment of fungal respiratory diseases.

## MATERIALS AND METHODS

### Fungal culture

The *A. fumigatus* B5233 strain was gifted by K. J. Kwon-Chung (National Institutes of Health [NIH]) and grown as previously described (18). Briefly, *A. fumigatus* was cultured at 37°C for 3–5 days on glucose minimal medial slants. To harvest conidia, a sterile solution of deionized water with 0.01% Tween 20 was added to each slant, and the surface was gently agitated with a sterile cotton swab. The resulting suspension was filtered through a 40 µm cell strainer to remove hyphal fragments. The conidia were then washed three times with sterile phosphate-buffered saline (PBS) and counted using a LUNA automated cell counter. Conidia were used immediately and applied to the apical surface in HBSS media (total volume of 400 µL) (StemCell, #37150).

The *C. posadasii* Silveira strain was received from BEI (NR-48944). WT *C. posadasii* was plated on 2× GYE media [20 g D-(+)-glucose (Sigma-Aldrich, Cat#G5767), 10 g Bacto Yeast Extract (ThermoFisher, Cat#212750), and 15 g Bacteriological agar (Sigma-Aldrich, Cat#A5306), in 1 L of diH_2_O]. Cultures were grown at 30°C at ambient CO_2_ for 4–7 weeks with weekly observation. Arthroconidia were harvested using sterile 1× PBS (Corning, Cat# 21-040-CV) and agitated with a cell scraper (Corning, Cat# 3010). Suspension was passed through a 40 µm cell strainer (CellTreat, Cat#229481), vortexed, and centrifuged at 12,000 × *g* for 8 min. The pellet was resuspended in PBS and washed twice. Arthroconidia were used immediately and applied to the apical surface in HBSS media (total volume of 400 µL).

### Isolation and differentiation of hAECs

The anatomical source of human airway basal cells was small airways (bronchioles). Primary hAECs were cultured following the established protocols (17, 18). Basal cells were maintained in SAGM (PromoCell, #C-21170), supplemented with 5 µM Y-27632 (Tocris, #1254), 1 µM A-83-01 (Tocris, #2939), 0.2 µM DMH-1 (Selleck Chemicals, #S7146), 0.5 µM CHIR99021 (Tocris, #4423), and 1% penicillin/streptomycin (Gibco, #151410122) on plates coated with laminin-enriched 804G-conditioned media. For differentiation into a pseudostratified epithelium at the ALI, the apical compartments of 12 mm Transwell inserts with 0.4 µm pore polyester membranes (Corning, #3460) were pre-coated with 804G-conditioned media for at least 4 hours. After removing the coating media, a suspension of basal cells in SAGM was applied to the apical compartment, and SAGM was added to the basolateral compartment for overnight incubation. The next day, SAGM was replaced with a 1:1 mixture of PneumaCult-ALI medium (StemCell, #05001) and DMEM/F-12 (Gibco, #11320033) for further overnight incubation. The medium in the apical compartment was then removed to establish the ALI. The hAECs were maintained at ALI for 16–23 days, with the apical compartment kept dry and the basolateral medium refreshed regularly. To ensure an intact epithelium, we measured the transepithelial electrical resistance (TEER) on the day of the experiment. All epithelium demonstrated a TEER reading of at least 1,000 ohms. All studies used a technical triplicate from a single donor.

### Staining and quantification of cells in ALI cultures

The staining and quantification were performed similarly to (26). Briefly, the luminal side of the ALI membranes was washed with 200 µL of PBS or 10 mM DTT (Thermo Fisher Scientific R0861) in PBS for 1 min, followed by an additional wash with PBS. ALI membranes were fixed in 4% paraformaldehyde for 15 min and washed in PBS for 5 min. ALI membranes were preserved in PBS until staining. To stain, ALI membranes were permeabilized in PBS-0.3% Triton X-100 (PBST) for 15 min. Samples were stained with primary antibody at 4°C overnight, diluted in 3% BSA, 0.5% in PBST. Samples were stained with secondary antibodies at room temperature for 1 h, diluted in 3%BSA in PBST. The primary and secondary antibodies used and their concentrations are listed in Table S2. The ALI membranes were then mounted using a mounting medium containing DAPI (Southern Biotech DAPI Fluoromount G 0100-01). Confocal images were obtained using an Olympus FV10i confocal laser-scanning microscope with a 60× objective, and the images were processed using Image J, and cells from the ALI membrane were manually counted. Three different basal cell isolations from three separate regions of a single human donor were assessed and averaged.

### Infection of hAECs and isolation of single cells

*A. fumigatus* B5233 or *C. posadasii* Silveira strain arthroconidia (10^7^/cm^2^) were added to the apical side of the hAEC Transwells for 6 or 18 h, respectively. Once infection was complete, all ALI media was aspirated and hAECs were washed with ice-cold wash buffer (1,000 µL basolateral, 500 µL apical; 10% heat-inactivated fetal bovine serum [Gibco] in PBS). The wash buffer was pipetted up and down several times to liberate non-adherent particles and repeated twice. After three total washes, the Transwells were transferred to a 50 mL conical containing 6 mL of dissociation solution (TrypLE [Thermo Fisher, #1264013]) and incubated on a rocking platform at 37°C. Every 3–5 minutes, conical tubes were vortexed to break up clumps. Once the Transwell had turned clear, dissociation was complete, and the reaction was quenched using one volume of wash buffer. Samples were then passed through a 40 µm filter to remove any large clumps before being spun down at 42 × *g* for 5 min. Cells were then resuspended in fresh wash buffer, and viability/counts were attained using Trypan Blue (*A. fumigatus* experiment) or acridine orange (*C. posadasii* experiment) and a LUNA automated cell counter. Viability was >90%.

### Single-cell RNA sequencing and library preparation

Single-cell suspensions from infected and mock hAECs were loaded into the Chromium Controller (10× Genomics) for droplet generation. For each sample, 16,000 cells were loaded per channel, aiming for a recovery of 10,000 single cells. There was only one channel per condition. The scRNA-seq libraries were constructed using the Chromium Next GEM single cell 3′ V3.1 Reagent Kit (10× Genomics, PN 1000268). Library quality was assessed with an Agilent 2100 Bioanalyzer and TapeStation. All gene expression libraries were multiplexed and sequenced at the Harvard Biopolymers Core Facility at a depth of 9,751 reads/cell for *A. fumigatus* infected, 11,059 reads/cell for *A. fumigatus* mock, 6,180 reads/cell for *C. posadasii* infected, and 6,531 reads/cell for *C. posadasii* mock on an Illumina Nextseq 500/550 instrument using the high-output v2.5 75 cycles kit with the following sequencing parameters: read 1 = 26; read 2 = 56; index 1 = 8; and index 2 = 0. Demultiplexing the sequence reads to create FASTQ files and alignment to the human genome reference GRCh38 (version refdata-gex-GRCh38-2020-A, 10× Genomics) were performed using Cell Ranger (version 7.1.0, 10× Genomics) commands mkfastq and count, respectively, and subsequent analysis was performed to evaluate transcriptional changes.

### Data analysis

Quality filtering, variable gene selection, and clustering were performed as described previously (77). We performed unsupervised clustering using the Leiden algorithm (leidenAlg v1.1.3) and annotated clusters based on canonical markers: KRT5 (basal), SCGB1A1, AZGP1 (secretory), MUC5AC (goblet), FOXJ1 (ciliated), CALCA, FOXI1, TRPM5 (rare types), KRT13 (hillock), MKI67 (proliferating), and MT1G, ICAM1. Clusters with overlapping markers were merged. Analysis was performed in R (v4.2.1) using Seurat (v5.0.3), harmony (v1.2.0), and leidenAlg (v1.1.3).

For each cell, we quantified the number of genes for which at least one read was mapped and then excluded all cells with fewer than 800 or greater than 10,000. We also excluded cells in which more than 30% of transcripts mapped to the mitochondrial genome. Expression values *E_i_*_,_*_j_* for gene *i* in cell *j* were calculated by dividing UMI count values for gene *i* by the sum of the UMI counts in cell *j*, to normalize for differences in coverage, and then multiplying by 10,000 to create TPM-like values, and finally calculating log_2_(TPM + 1) values.

For each gene, we modeled the relationship between detection fraction (proportion of cells in which at least one UMI was observed) and the log of the total number of UMIs using logistic regression. Outliers from this curve are expressed in a lower fraction of cells than would be expected and are thus highly variable; that is, they are specific to a cell type, treatment, condition, or state. We selected the top 3,000 genes with the highest residuals as highly variable genes. We restricted the expression matrix to the subsets of variable genes and high-quality cells noted above, and values were centered and scaled before input to PCA. For *C. posadasii*, data from different conditions were then integrated using the Harmony algorithm (78), before shared nearest-neighbor network construction and clustering using the Leiden algorithm as we have described previously (79). Differential expression (DE) tests were performed using MAST (80). All DE tests were run by comparing all cells of each type between conditions. For each cell type, genes were only tested if they were detected in greater than 10 cells. Chemokines were selected using the HUGO gene set chemokine ligands (group 483, https://www.genenames.org/data/genegroup/#!/group/483). Pathway enrichment was performed using the “EnrichR” R package.

## Data Availability

The raw data associated with scRNA-seq studies are available in the GEO database at GSE275378.

## SUPPLEMENTAL MATERIAL

Fig. S1 mbio.02121-25-s0001.tif UMAP of rare cells.

Legend mbio.02121-25-s0002.docx Legend for Fig. S1.

Table S1 and S2 captions mbio.02121-25-s0003.docx Captions for supplemental tables.

Table S1 mbio.02121-25-s0004.xlsx Downregulated genes.

Table S2 mbio.02121-25-s0005.xlsx Antibodies used.

ASM does not own the copyrights to Supplemental Material that may be linked to, or accessed through, an article. The authors have granted ASM a non-exclusive, world-wide license to publish the Supplemental Material files. Please contact the corresponding author directly for reuse.
