# Supplementary figures and images for: Single-cell analysis of human airway epithelium identifies cell-type-specific responses to Aspergillus and Coccidioides
Source: mBio. 2025 Oct 13;16(11):e02121-25. doi: 10.1128/mbio.02121-25 (PMC12607908; doi:10.1128/mbio.02121-25)

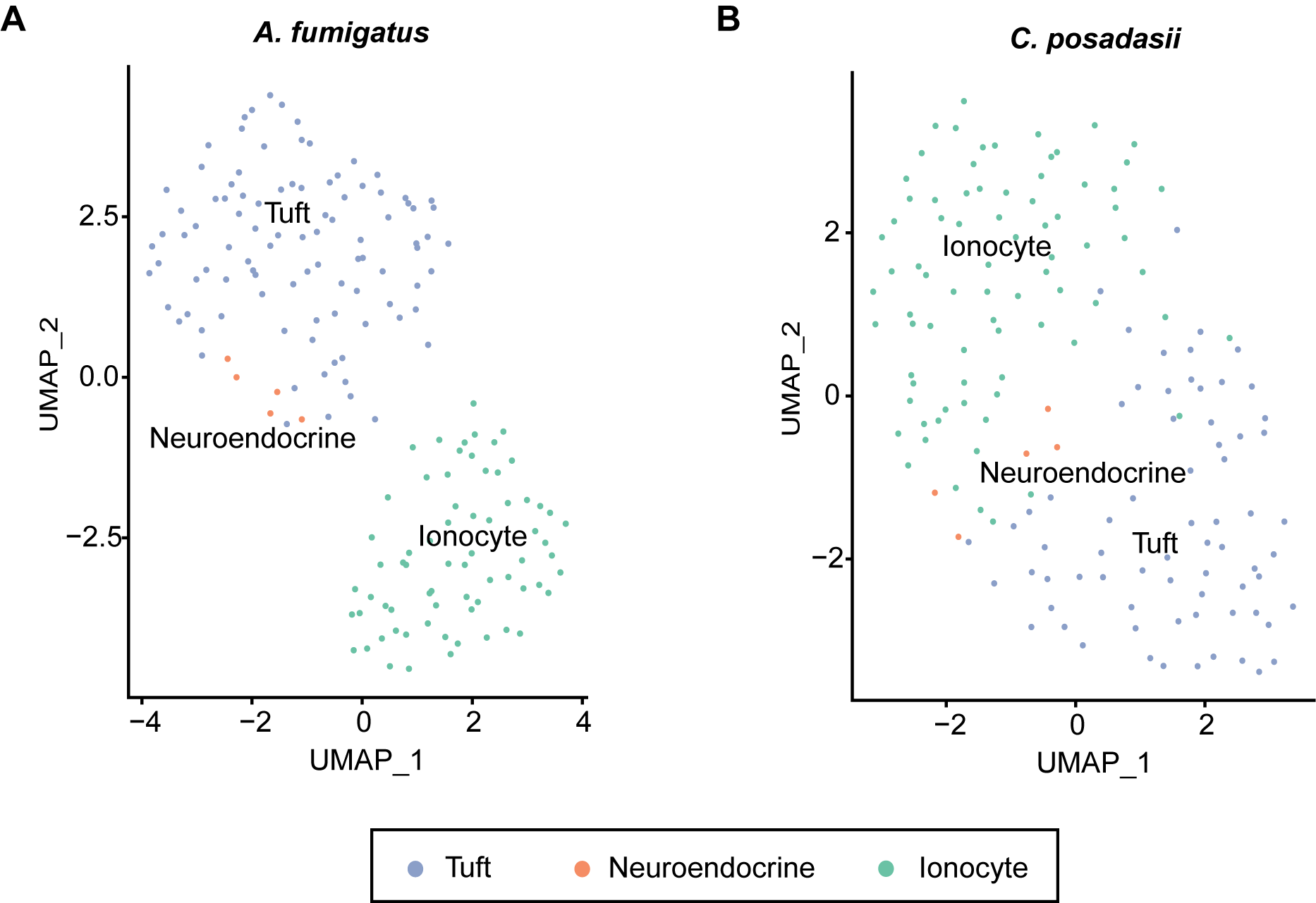

Supplement: Fig. S1 — UMAP of rare cells. [file mbio.02121-25-s0001.tif]
