# Supplementary material for: Single-cell analysis of human airway epithelium identifies cell-type-specific responses to Aspergillus and Coccidioides
Source: mBio. 2025 Oct 13;16(11):e02121-25. doi: 10.1128/mbio.02121-25 (PMC12607908; doi:10.1128/mbio.02121-25)
Supplement: Legend — for Fig. S1. [file mbio.02121-25-s0002.docx]

**Supplemental Figure 1**: UMAP of rare cells (Tuft, Neuroendocrine and Ionocytes) in hAECs when stimulated by either (A) *A. fumigatus* or (B) *C. posadasii*.
