# Supplementary material for: Single-cell analysis of human airway epithelium identifies cell-type-specific responses to Aspergillus and Coccidioides
Source: mBio. 2025 Oct 13;16(11):e02121-25. doi: 10.1128/mbio.02121-25 (PMC12607908; doi:10.1128/mbio.02121-25)
Supplement: Table S1 and S2 captions — Captions for supplemental tables. [file mbio.02121-25-s0003.docx]

**Supplemental Table 1**: List of downregulated genes in hAECs stimulated by *A. fumigatus*. Note that none of these genes were statistically downregulated.

**Supplemental Table 2**: List of antibodies used in this study.
